# Humanistic and Economic Burden of Spinal Muscular Atrophy in the Gulf Region

**DOI:** 10.7759/cureus.114046

**Published:** 2026-08-06

**Authors:** Baher Elezbawy, Sherif Abaza, Amal Alnajjar, Hana Alabdulkarim, Hussain A Al-Omar, Sahar Fahmy, Sara Aldallal, Tamás Ágh, Ahmad Fasseeh

**Affiliations:** 1 Evidence Synthesis, Syreon Middle East, Alexandria, EGY; 2 Doctoral School of Pharmaceutical Sciences, Semmelweis University, Budapest, HUN; 3 Health Economics, Syreon Middle East, Alexandria, EGY; 4 Drug and Poison Information Center, Security Forces Hospital, Riyadh, SAU; 5 Doctoral School of Applied Informatics and Applied Mathematics, Óbuda University, Budapest, HUN; 6 King Abdullah International Medical Research Center, Ministry of National Guard Health Affairs, Riyadh, SAU; 7 Clinical Pharmacy, College of Pharmacy, King Saud University, Riyadh, SAU; 8 Undersecretary Office, Department of Health, Abu Dhabi, ARE; 9 Health Economics, Emirates Health Economics Society, Dubai, ARE; 10 Health Economics, Syreon Research Institute, Budapest, HUN; 11 Modelling, Syreon Middle East, Alexandria, EGY

**Keywords:** disease burden, economic burden, humanistic burden, productivity lost, resource utilization, spinal muscular atrophy

## Abstract

Background: Spinal muscular atrophy (SMA) is a severe, fatal genetic disorder. This study aimed to quantify the humanistic and economic burden of SMA in four Gulf countries, the Kingdom of Saudi Arabia (KSA), the United Arab Emirates (UAE), Oman, and Bahrain, to provide decision-makers with evidence-based strategies for burden mitigation.

Methods: A burden of disease model was developed to quantify SMA's humanistic burden via disability-adjusted life years (DALYs) and its economic burden through direct healthcare costs and indirect productivity losses experienced by patients and caregivers.

Results: Type 1 SMA caused the highest burden across all countries, followed by types 2 and 3. Total DALYs were 274,008 in KSA, 35,877 in UAE, 33,459 in Oman, and 8,679 in Bahrain. National direct medical costs totaled 114.9 million USD, 25.0 million USD, 9.5 million USD, and 3.8 million USD in KSA, UAE, Oman, and Bahrain, respectively. Annual indirect costs, driven by productivity losses among patients and caregivers, were estimated at 28.1 million USD in KSA, 5.3 million USD in UAE, 3.9 million USD in Oman, and 0.99 million USD in Bahrain.

Conclusions: The burden of SMA is considerable, particularly in the more severe phenotypes, with a profound humanistic impact and high medical costs. While results vary by population size, the consistent patterns across countries underscore the disease's severity and the urgent need for effective mitigation strategies.

## Introduction

Spinal muscular atrophy (SMA) is a rare genetic neuromuscular disorder that severely affects patients’ survival and quality of life (QoL) [1]. The epidemiology of SMA is not well established based on the available evidence [1]; however, the most comprehensive study to date, conducted by Verhaart et al. in 2017, estimated the prevalence to be 1-2 patients per 100,000 persons and the incidence to be 1 in 10,000 live births [2]. Actual prevalence and incidence values might differ by country; however, the global estimates are expected to be close to this study’s estimates [2]. Although rare, SMA imposes a tremendous economic burden on patients, health systems, and society due to its high mortality, severe morbidity, and cost of care [3,4].

SMA is classified into five subtypes (0 to 4), based on onset and motor milestones [1]. Type 0 is an ultra-rare, prenatal-onset form that is typically fatal within months, while Type 1 involves symptom onset before six months with severe atrophy and a life expectancy often under two years. Type 2 onset occurs between 6 and 12 months, allowing for survival into adulthood despite significant support requirements. Type 3 is diagnosed after 18 months and Type 4 in adulthood; both phenotypes are characterized by milder motor impairment and a generally normal life expectancy [1].

SMA burden is disproportionately high due to its severe prognosis across the various subtypes [5]. Decision-makers in any country require data to make evidence-informed decisions on resource allocation and reimbursement, especially for high-burden diseases [6]. Burden of disease studies enable decision-makers to understand the impact of a disease and allocate their resources more efficiently [6]. They typically address three key domains: clinical burden, humanistic burden, and economic burden. The clinical burden focuses on the morbidity and mortality of the disease. The humanistic burden focuses on the negative impact of the disease on QoL. The economic burden considers the direct and indirect costs incurred by patients, caregivers, society, or health systems as a whole [7,8].

The clinical burden of SMA is well documented in the literature, showing the average survival associated with each type, as well as the clinical manifestations and comorbidities [1,4,9]. Clinical burden per patient generally does not vary significantly between countries, as patients worldwide experience similar manifestations and complications [10,11]. In contrast, the humanistic and economic burden components can vary significantly between countries due to differences in population size, economic status, healthcare services, service costs, and other contextual factors. SMA severely affects patients and caregivers’ QoL [12] and imposes a substantial financial burden on families and society for its medical costs as well as indirect costs [3].

While some studies have quantified the humanistic and economic aspects in certain countries [10,11], no such studies exist in some of the Gulf Cooperation Council (GCC) countries, particularly in Bahrain, Oman, and the United Arab Emirates (UAE), leaving decision-makers in those countries without the necessary data to make evidence-informed decisions for resource allocation.

Limited data are available on the burden of SMA in the GCC countries. A recent study in the Kingdom of Saudi Arabia (KSA) reported the socioeconomic burden of SMA through the implementation of a caregiver questionnaire [13]. The findings showed that Type 1 was the most severe and resource-intensive form, whereas Type 3 patients had a better QoL, but incurred the highest out-of-pocket payments. The study also provided detailed QoL data for SMA Type 1-4 patients, emphasizing that QoL improves with later-onset types. Although SMA has been studied in some studies from Bahrain, Oman, and the UAE [14-17], none have quantified specific burden of disease components.

The aim of this study is to quantify the humanistic and economic burden of SMA in four major GCC countries, namely, KSA, UAE, Oman, and Bahrain. The study aims to estimate the number of SMA patients in each of these countries, assess how SMA affects QoL (humanistic burden), and quantify both direct and indirect costs associated with SMA in each country. The findings of this research are expected to support local decision-makers by providing a clearer understanding of the true burden of the disease, thereby enabling more informed and evidence-based resource allocation decisions.

## Materials and methods

A burden of disease model was developed to quantify both the humanistic and economic burden of SMA, tailored to each country’s specific context. A bottom-up approach was applied, starting with detailed estimates of each burden component on a per-patient basis in each country. These per-patient values were then multiplied by the estimated patient population in each country, providing estimates for both burden per patient and burden per country.

The study followed a sequential process, starting with a literature search to understand the disease area, assess available data, and review similar studies to inform the model design. The model was then built and refined to capture all components and benefit from the available extracted data. Local data collection was undertaken to populate the model with inputs derived from multiple sources, including published literature, and relevant organizational websites from each country. Additionally, expert interviews were conducted in each country to estimate the resource utilization patterns for calculating the direct costs of the disease.

Once the initial models were constructed, their results were validated through meetings with local experts in each country. Expert feedback and recommendations were incorporated to refine the model, improve its robustness, and ensure its local relevance. The final models were then analyzed to generate detailed burden estimates for each country.

Because SMA patients’ prognosis, severity, and QoL vary significantly by type, all burden of disease estimates were calculated separately for each SMA type. Data inputs and calculated estimates were adjusted to reflect the year 2026. Due to their low prevalence and limited representation in the literature [18], Types 0 and 4 were considered to have a negligible burden compared to Types 1, 2, and 3. Accordingly, results are reported by type and as a combined estimate for Types 1-3, excluding Types 0 and 4 SMA.

Estimated number of patients

To estimate the number of patients in each country, we used a previously developed global prevalence model that estimated the number of patients in each country in 2026, reflecting the diseases' point prevalence by SMA subtype [19]. Table 1 shows the estimated number of patients in 2026 in each country.

**Table 1:** Estimated number of SMA patients in each country in 2026. KSA: Kingdom of Saudi Arabia; SMA: spinal muscular atrophy; UAE: United Arab Emirates.

SMA type	KSA	UAE	Oman	Bahrain
Type 1	861	134	114	29
Type 2	1,983	256	250	64
Type 3	1,278	146	159	41
Total	4,122	536	523	134

Humanistic burden

The humanistic burden of SMA was estimated through calculating disability-adjusted life years (DALYs). For each country, DALYs were calculated separately for each SMA type by adding the years of life lost (YLL) to the years lived with disability (YLD), as shown in the following formula:

$\text{DALYs = YLL + YLD}$

Years of Life Lost

To estimate YLL for each country, we subtracted the average survival of SMA patients from the average life expectancy of the general population in that country, thereby determining the average YLL due to the disease. YLL was calculated using the formula:

$\mathrm{YLL} = \text{General population average life expectancy in each country} - \text{average survival of SMA patients (by subtype)}$

For the general population life expectancy, we used the average life expectancy values for each country provided by the United Nations World Population Prospects for 2026 [20].

For the mean survival of SMA patients, we used data derived from a published survival model based on an individual patient data-enhanced methodology [21]. Using this model, restricted mean survival was calculated by integrating disease-specific mortality with background mortality from the general population. Mean survival, representing the life expectancy of SMA patients, was estimated by calculating the area under the curve of each SMA subtype. The resulting mean survival estimates were 7.39 years for patients with SMA Type 1 and 36.70 years for those with SMA Type 2. Evidence from a systematic literature review indicates that SMA does not appear to significantly affect survival among patients with Type 3 [12]. Therefore, these patients were assigned the life expectancy of the general population and were not assigned any YLL due to early mortality.

Years Lived With Disability

To estimate YLD per patient, we multiplied the loss in QoL (disutility) of each patient by the number of years they were expected to live with this disability (SMA survival). YLD was calculated using the formula:

$\text{YLD = Disutility value (per patient)} \times \text{average survival of SMA patients (for each subtype)}$

Disutility values for different SMA subtypes were not consistently or robustly reported in the literature. Therefore, a proxy approach was used to estimate the disutility. A systematic literature review on the humanistic and clinical burden of SMA reported average values from the Health Utilities Index Mark 3 (HUI3) questionnaire for each SMA subtype [12]. The HUI3 is a validated preference-based instrument that measures health-related QoL on a scale from 0 (worst health state) to 1 (perfect health), comparable to utility values commonly used in health economic evaluations [22]. The reported HUI3 values were used to estimate the disutility for each subtype using the formula:

$\mathrm{Disutility} = 1 - \text{reported HUI3 value}$

HUI3 values were selected rather than other QoL measures reported in the review because they were available for all relevant SMA subtypes. This approach was intended to provide an approximate estimate of the QoL loss associated with each SMA subtype. However, uncertainty remains because the systematic review reported substantial heterogeneity among the included studies.

To explore the potential impact of this uncertainty, two additional scenario analyses were conducted to capture a range of burden estimates and ensure that the true burden lies within these estimates. In Scenario 1, utility values for each subtype were increased by 50% relative to those reported in the systematic review, representing a lower burden scenario. In Scenario 2, utility values were reduced by 50%, representing a higher burden scenario. Finally, DALYs were calculated based on these utility values using the three scenarios. The reported utility values and the calculated scenarios are shown in Appendix A.

Economic burden

Indirect Costs

Indirect costs were calculated for patients and caregivers based on the time they lost due to the disease. The following formula was used to estimate the indirect costs:

$\begin{aligned} \text{Patient/caregiver indirect cost} \;=\;& \text{Number of adult patients of each type} \times \text{labor force participation rate} \\ &\times \text{employment rate} \times \text{hourly wage} \times \text{hours lost} \end{aligned}$

Indirect costs were assessed exclusively for patients aged 16 and older, as individuals younger than 16 years are generally not part of the productive workforce. Consequently, younger patients were excluded from the productivity loss analysis.

For caregiver-related productivity losses, it was assumed that each patient requires the support of one primary caregiver. Consequently, caregiver burden was estimated based on the total number of SMA patients, including both adults and children, as patients with SMA typically require continuous assistance regardless of age.

Indirect cost calculations were adjusted according to the labor force participation rate in each country to account for the proportion of the population that is not actively engaged in the workforce, thereby avoiding overestimation of productivity losses.

The number of hours lost for productivity by patients and their caregivers were derived from a systematic literature review that reported estimates of time lost due to SMA-related disability and caregiving responsibilities [12]. A summary of the identified estimates for productivity losses among patients and caregivers is presented in Appendix B. Country-specific hourly wage data were obtained from an online database reporting salaries in the included countries [23].

Direct Medical Costs

Direct medical costs were estimated using country-specific costing data for each of the four GCC countries included in the analysis. Resource utilization patterns were collected from local expert interviews, and unit costs of medications and healthcare services were collected from publicly available national databases and local hospital pricelists.

To estimate healthcare resource utilization, a structured questionnaire was developed and administered to clinical experts in each country during online interviews. To create the questionnaire, we searched published studies to identify relevant cost components associated with the management of SMA [3,18,24]. These components included, but were not limited to, diagnostic tests, routine monitoring procedures, management of complications, and types of medical visits. Experts were also encouraged to identify and report any additional cost components that might not have been captured in the initial questionnaire to ensure comprehensive coverage of all relevant healthcare resources.

Before starting the interviews, the questionnaire was reviewed and validated by a clinical expert specialized in the management of SMA to ensure that all clinically relevant aspects of the disease and its management were adequately captured. The questionnaire included three sections: (1) diagnostics costs, (2) monitoring costs, and (3) medical management costs.

Following questionnaire development and validation, expert interviews were conducted to estimate the frequency of resource utilization for each cost component (e.g., number of outpatient visits per year by SMA subtype, frequency of laboratory tests, and other medical services). In each country, two clinical experts actively involved in the treatment of SMA patients participated in the interviews. The average of their reported estimates was used in the analysis to minimize potential bias arising from individual clinical practice variations.

Given the rarity of SMA and the limited number of clinical experts specializing in its management, experts were recruited using convenience sampling. Clinical experts were eligible to participate if they were currently treating SMA patients within the respective country and had at least three years of continuous experience in managing SMA cases.

Unit costs were obtained from local sources, including published national drug lists [25-28], hospital price lists, and insurance price lists. Costs were adjusted to purchase prices to represent the actual economic burden paid by the payer or insurer.

The average annual direct medical cost per patient for each SMA subtype was calculated using the following formula:

$\mathrm{Cost}_{\mathrm{Subtype}} = \sum (P_i \times F_i \times C_i)$

*P_i_* = proportion of patients utilizing cost item *i*, *F_i _*= annual frequency of utilization of cost item *i*, *C_i _*= unit cost of cost item *i*.

The average cost per patient across all SMA subtypes was estimated as a weighted average based on the distribution of SMA subtypes in the population:

$\text{Average cost per patient} = (\mathrm{Prop}_{\mathrm{Type1}} \times \mathrm{Cost}_{\mathrm{Type1}}) + (\mathrm{Prop}_{\mathrm{Type2}} \times \mathrm{Cost}_{\mathrm{Type2}}) + (\mathrm{Prop}_{\mathrm{Type3}} \times \mathrm{Cost}_{\mathrm{Type3}})$

Finally, the total direct medical cost at the population level was estimated by multiplying the number of patients in each subtype by the corresponding average cost:

$\text{Total population cost} = (N_{\mathrm{Type1}} \times \mathrm{Cost}_{\mathrm{Type1}}) + (N_{\mathrm{Type2}} \times \mathrm{Cost}_{\mathrm{Type2}}) + (N_{\mathrm{Type3}} \times \mathrm{Cost}_{\mathrm{Type3}})$

The full questionnaire used for data collection is provided in Appendix C.

Total estimated burden

To estimate the overall burden of SMA at the country level, all cost components were aggregated. The economic burden included both direct medical costs and indirect costs related to productivity losses. In addition, the humanistic burden of the disease was quantified. To express this burden in monetary values, DALYs lost at the population level were multiplied by the gross domestic product (GDP) per capita of the respective country. Under this approach, each DALY lost, representing one year of healthy life lost due to premature mortality, disability, or both, was assigned a monetary value equivalent to one time the GDP per capita [29]. This valuation method does not represent actual financial losses but rather provides a hypothetical economic valuation of health loss, allowing the humanistic burden to be expressed in monetary terms and compared with the economic costs of the disease.

Finally, the total burden of SMA in each country was estimated by combining the three components; direct medical costs, indirect costs, and the monetized value of DALYs lost (representing the humanistic burden).

Data processing and analysis

Mean survival was used instead of median survival to better capture the full distribution of survival outcomes in SMA. Given the highly skewed survival patterns, particularly in Type 1 SMA, where a small proportion of patients survive substantially longer, mean survival provides a more comprehensive estimate for modelling disease burden.

To address the uncertainty in YLD estimation, minimum and maximum HUI values were calculated, and corresponding ranges were reported for YLDs and DALYs. Cost data were initially collected in local currencies for each country and subsequently converted to US dollars (USD) using 2026 exchange rates as reported by the World Bank [30]. No discounting was applied to costs because direct and indirect costs were estimated on an annual basis for the projected 2026 prevalent population.

All analyses were conducted using Microsoft Excel, version 2606 (Microsoft Corporation, Redmond, WA, USA). Mean values for resource utilization were calculated for multiple experts’ inputs to minimize individual bias. In cases where substantial discrepancies were identified (defined as one estimate being at least double another), follow-up discussions were conducted with the experts to clarify assumptions and reach a consensus on the final values.

## Results

The study findings present estimates of the humanistic and economic burden of SMA at both the individual and population levels across the included countries. Per-patient estimates represent the average burden experienced by an individual living with SMA within each country, while population-level estimates account for the projected number of patients based on the estimated point prevalence for 2026.

Humanistic burden

Patient Humanistic Burden

SMA imposes a humanistic burden on patients across all countries included in the study. For SMA Type 1, a patient loses approximately 72-76 years of life expectancy compared to the general population in his/her respective country. In addition, disability contributes to approximately 6 YLDs, ranging from six to seven years based on the base HUI-3 utility estimates and ±50% uncertainty adjustments. Consequently, the total burden for Type 1 patients ranges between 78 and 82 DALYs per patient.

For SMA Type 2 patients, the estimated YLL ranges from 43 to 47 years across the countries included in the analysis. However, these patients live longer with disease-related disability, resulting in substantial YLD estimated at 31 years, with a range of 28-34 years when applying HUI-3 base estimates and ±50% uncertainty adjustments. Combined, the overall burden for Type 2 patients reaches approximately 74-78 DALYs per patient.

For SMA Type 3, patients experience approximately 39 YLD, resulting in 39 DALYs per patient. The uncertainty range is wider (11-56 DALYs) due to greater variability in QoL estimates over the prolonged disease duration experienced by these patients. The detailed estimates of YLL, YLD, and DALYs per patient by SMA subtype and country are presented in Table 2.

**Table 2:** The humanistic burden of SMA per patient in each country. YLL: years of life lost; YLD: years lived with disability; DALYs: disability-adjusted life years; KSA: Kingdom of Saudi Arabia; SMA: spinal muscular atrophy; UAE: United Arab Emirates; HUI-3: Health Utilities Index Mark 3; QoL: quality of life. ^*^Values within brackets represent the range of uncertainty when using HUI-3 lower and upper estimates to estimate the effect of SMA on QoL.

SMA type	Humanistic burden component	KSA	UAE	Oman	Bahrain
Type 1	YLL	76	74	72	73
	YLD*	6 (6-7)	6 (6-7)	6 (6-7)	6 (6-7)
	DALYs (YLL + YLD)*	82 (82-83)	81 (80-81)	78 (78-79)	80 (79-80)
Type 2	YLL	47	45	43	44
	YLD*	31 (28-34)	31 (28-34)	31 (28-34)	31 (28-34)
	DALYs (YLL + YLD)*	78 (75-80)	76 (73-79)	74 (71-76)	75 (72-78)
Type 3	YLL	0	0	0	0
	YLD*	39 (22-56)	39 (22-56)	39 (11-56)	39 (22-56)
	DALYs (YLL + YLD)*	39 (22-56)	39 (22-56)	39 (11-56)	39 (22-56)

Population Humanistic Burden

**Figure 1:**
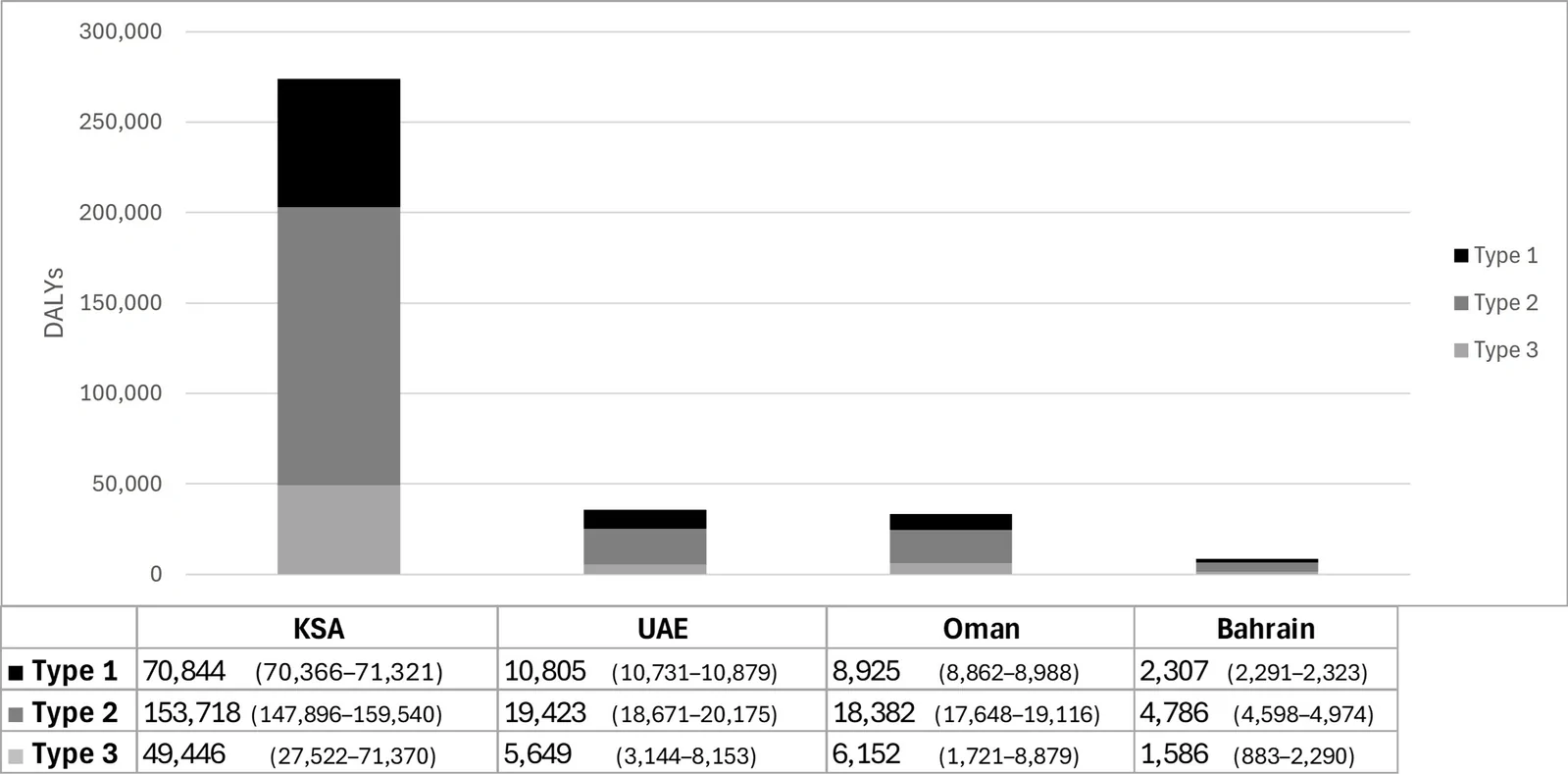
DALYs lost per population in each country. DALYs: Disability-adjusted Life Years, KSA: Kingdom of Saudi Arabia, UAE: United Arab Emirates; SMA: spinal muscular atrophy; QoL: quality of life; HUI-3: Health Utilities Index Mark 3. Values within brackets represent the range of uncertainty when using HUI-3 lower and upper estimates to estimate the effect of SMA on QoL.

For the overall SMA population in each country, DALYs reached 274,008 in KSA, 35,877 in UAE, 33,459 in Oman, and 8,679 in Bahrain. Uncertainty ranges represented by the lower and upper estimates derived from the HUI-3 utility analysis appear wider at the population level due to the amplified effect when uncertainty in a single patient is extended to the whole population. The population-level DALY estimates for each SMA subtype and country are presented in Figure 1.

Economic burden

The economic burden of SMA comprises two main components: indirect costs, represented by productivity losses experienced by patients and their caregivers, and direct medical costs of the disease representing the healthcare resources required for disease management.

Indirect Costs

The indirect costs across the included countries show a consistent trend, with caregiver burden being highest in Type 1 and decreasing toward Type 3, reflecting the natural progression and severity of the disease. Productivity losses among caregivers represent the major component of the indirect costs, especially in Type 1 and Type 2 SMA patients.

SMA Type 2 patients sometimes survive into adulthood, but caregiver involvement remains significant throughout the disease course. Consequently, caregiver productivity losses continue to represent the largest share of indirect costs, although some productivity losses among patients may also occur.

SMA Type 3 patients typically reach adulthood and may participate in the workforce. As a result, productivity losses among patients themselves become a more substantial component of indirect costs. While caregiver assistance is still required, a proportion of the productivity burden shifts from caregivers to patients compared with the more severe SMA subtypes.

Table 3 summarizes the estimated annual indirect costs of SMA across the countries included. The total indirect burden was estimated at 28.1 million USD in KSA, 5.3 million USD in UAE, 3.9 million USD in Oman, and 988 thousand USD in Bahrain.

**Table 3:** Annual indirect costs of SMA represented in productivity loss due to the disease. KSA: Kingdom of Saudi Arabia; SMA: spinal muscular atrophy; UAE: United Arab Emirates.

SMA type	Indirect cost component	KSA (USD)		UAE (USD)	Oman (USD)	Bahrain (USD)
Type 1	Patients		-	-	-	-
	Caregivers		5,875,058	1,297,961	763,301	197,992
	Total cost (patients and caregivers)		5,875,058	1,297,961	763,301	197,992
Type 2	Patients		1,462,587	268,670	205,087	50,887
	Caregivers		14,781,651	2,715,318	2,072,710	514,290
	Total cost (patients and caregivers)		16,244,238	2,983,988	2,277,797	565,177
Type 3	Patients		2,393,558	399,729	347,109	89,400
	Caregivers		3,630,906	606,369	526,546	135,615
	Total cost (patients and caregivers)		6,024,465	1,006,098	873,655	225,014
All types combined	Total cost (patients and caregivers)		28,143,761	5,288,047	3,914,752	988,183

Direct Medical Costs

Direct costs per patient: Annual direct medical costs per patient were highest for Type 1, followed by Type 2 and Type 3 SMA across all countries included in the analysis. This pattern reflects the greater clinical severity and healthcare needs associated with early-onset SMA.

For Type 1 patients, hospitalization costs represented the largest share of total direct medical costs, reflecting the intensive medical care and frequent hospital admissions required for disease management. As disease severity decreases across subtypes, the relative contribution of hospitalization costs declines. In Type 2 patients, hospitalization still represents a considerable portion of total costs but to a lesser extent than in Type 1. In Type 3 patients, hospitalization accounts for only a small proportion of the total direct cost, with most expenditures attributed to other healthcare resource utilization such as outpatient care, and supportive treatments. This pattern was consistently observed across all four countries.

Appendix D shows the detailed annual direct costs per patient by SMA subtype and country. In KSA, the annual direct cost per patient was estimated at 53,556 USD for Type 1, 25,955 USD for Type 2, and 13,536 USD for Type 3. In the UAE, the corresponding annual costs were 82,692 USD, 47,688 USD, and 11,443 USD for Types 1, 2, and 3, respectively. In Oman, the annual costs were estimated at 36,720 USD for Type 1, 21,168 USD for Type 2, and 5,886 USD for Type 3. Similarly, in Bahrain, annual direct medical costs per patient were 41,215 USD for Type 1, 33,710 USD for Type 2, and 11,362 USD for Type 3.

Direct cost for the population: When estimating the direct medical costs at the population level, the number of patients in each SMA subtype was incorporated into the calculations. As a result, the total costs associated with SMA Type 1 and Type 2 become relatively similar, despite the higher per-patient cost of Type 1, because Type 2 patients are more prevalent in the population. Consequently, the higher prevalence of Type 2 patients offsets the higher individual costs in Type 1. In contrast, SMA Type 3 contributes a smaller proportion of the overall direct medical burden.

Overall, the total annual direct medical cost of SMA at the national level was estimated at 114.9 million USD in KSA, 25.0 million USD in UAE, 9.5 million USD in Oman, and 3.8 million USD in Bahrain, as detailed in Table 4. The table summarizes direct costs, categorized into hospitalization and other resource utilization. The latter includes diagnostic procedures, routine monitoring (such as outpatient visits and laboratory tests), and outpatient care, as well as ongoing disease management costs, including symptomatic medications, physiotherapy, rehabilitation, medical aids, and respiratory care.

**Table 4:** Direct annual costs of the population in each country. SMA: spinal muscular atrophy; KSA: Kingdom of Saudi Arabia; UAE: United Arab Emirates.

SMA type	Direct cost component	KSA (USD)	UAE (USD)	Oman (USD)	Bahrain (USD)
Type 1	Hospitalization	23,953,788	9,045,119	3,365,743	1,051,481
	Other resource utilization	22,157,663	2,035,669	820,385	143,766
	Total cost	46,111,451	11,080,787	4,186,127	1,195,247
Type 2	Hospitalization	31,347,264	8,517,394	3,624,187	1,857,532
	Other resource utilization	20,120,576	3,690,722	760,494	300,085
	Total cost	51,467,840	12,208,115	4,384,681	2,157,617
Type 3	Hospitalization	4,977,810	682,036	552,055	360,713
	Other resource utilization	12,320,772	988,586	383,337	105,226
	Total cost	17,298,582	1,670,622	935,391	465,939
All types combined	Total cost (patients and caregivers)	114,877,872	24,959,525	9,506,200	3,818,803

Total estimated burden

The overall burden of SMA, including both economic and humanistic components, is summarized in Table 5. The table presents the breakdown of the total burden by SMA subtype and country, separating the economic burden (comprising direct medical costs and indirect productivity losses) from the humanistic burden, represented by the monetized value of DALYs lost.

**Table 5:** Total burden of SMA in each country. SMA: spinal muscular atrophy; KSA: Kingdom of Saudi Arabia; UAE: United Arab Emirates.

SMA type	Type of burden	Burden component	KSA (USD)	UAE (USD)	Oman (USD)	Bahrain (USD)
Type 1	Economic burden	Direct medical costs	46,111,451	11,080,787	4,186,127	1,195,247
		Indirect costs	5,875,058	1,297,961	763,301	197,992
	Humanistic burden	Monetary value of DALYs	336,540,215	73,471,822	24,488,042	9,252,068
	Total Type 1 burden	Economic + humanistic burden	388,526,723	85,850,571	29,437,471	10,645,308
Type 2	Economic burden	Direct medical costs	51,467,840	12,208,115	4,384,681	2,157,617
		Indirect costs	16,244,238	2,983,988	2,277,797	565,177
	Humanistic burden	Monetary value of DALYs	147,111,206	26,607,238	10,160,528	3,867,147
	Total Type 2 burden	Economic + humanistic burden	214,823,284	41,799,341	16,823,007	6,589,942
Type 3	Economic burden	Direct medical costs	17,298,582	1,670,622	935,391	465,939
		Indirect costs	6,024,465	1,006,098	873,655	225,014
	Humanistic burden	Monetary value of DALYs	23,789,307	3,890,164	1,709,436	644,372
	Total Type 3 burden	Economic + humanistic burden	47,112,354	6,566,884	3,518,482	1,335,325
All types combined	Economic burden	Direct medical costs	114,877,872	24,959,525	9,506,200	3,818,803
		Indirect costs	28,143,761	5,288,047	3,914,752	988,183
	Humanistic burden	Monetary value of DALYs	507,440,728	103,969,224	36,358,007	13,763,588
	Total burden all types	Economic + humanistic burden	650,462,361	134,216,796	49,778,959	18,570,574

Across all countries, the humanistic burden represents the largest share of the total burden, reflecting the significant loss of life expectancy and the substantial disability associated with SMA. This pattern is particularly evident in the more severe subtypes, where early disease onset leads to considerable years of life lost and prolonged periods lived with disability.

Overall, the total burden of SMA across all subtypes was estimated to reach approximately 650.5 million USD in KSA, 134.2 million USD in the UAE, 49.8 million USD in Oman, and 18.6 million USD in Bahrain. Within this total, the humanistic component accounted for the majority of the burden in each country, while the economic burden comprising direct medical costs and productivity losses represented a smaller but still substantial proportion.

## Discussion

SMA is a fatal disease associated with humanistic and economic burdens. The humanistic burden is driven by early mortality and disability, reflected in high DALYs, along with deterioration in the QoL of both patients and caregivers. The economic burden is also considerable, including high direct medical costs and indirect costs due to productivity losses. Given the predominantly pediatric onset, these impacts are particularly pronounced among caregivers.

Type 1 is associated with severe complications like respiratory failure and gastrostomy dependence, leading to resource-intensive complications. Type 2 requires lower immediate intensity but incurs high longitudinal costs due to prolonged survival, requiring mobility aids and orthopedic care. Types 3 and 4 are comparatively less severe; however, their near-normal life expectancy results in substantial cumulative burdens from outpatient monitoring, chronic medication, and caregiver support [1,3,5].

All four Gulf countries included in this study demonstrated a high burden of SMA. However, the per-patient burden varies due to differences in national characteristics such as GDP per capita, wages, labor force participation, and baseline QoL. At the population level, total burden is strongly influenced by population size, limiting direct comparisons between countries. For example, the higher burden observed in KSA likely reflects its larger population rather than a higher burden per patient.

The humanistic burden was highest in Type 1 SMA, followed by Types 2 and 3, consistent with differences in disease severity. On a per-patient basis, the humanistic burden showed little variation across countries, reflecting similar life expectancy and disease progression patterns throughout the region.

DALYs were highest in KSA, followed by the UAE, Oman, and Bahrain, driven by differences in population size. Despite being a rare disease, SMA contributes disproportionately to DALYs due to early mortality and severe disability.

At the subtype level, SMA Type 2 contributed the largest share of the humanistic burden across all countries, primarily due to its higher prevalence compared to other subtypes and its associated substantial disability.

At the population level, DALYs were highest in KSA, followed by the UAE, Oman, and Bahrain, reflecting the larger number of patients in countries with bigger populations. These findings highlight the burden of the disease. Although SMA is a rare condition, the relatively small number of patients contributes disproportionately to total DALYs due to early mortality in many cases and severe disability among survivors.

Regarding indirect costs, these were highest in the UAE, likely due to higher wage levels and the resulting greater productivity losses compared to the other included countries. Productivity losses among Type 1 patients are minimal, few survive into adulthood; however, their need for intensive, continuous care leads to considerable productivity losses for caregivers. Across all countries, Type 1 SMA accounted for the highest indirect costs, mainly driven by caregiver burden due to patients' full dependency. However, indirect costs contributed less to the overall burden than DALYs.

Direct costs were substantial across all countries. In Type 1 SMA, hospitalization was the primary cost driver, accounting for the majority of costs, while in Types 2 and 3, other healthcare resources such as rehabilitation and symptomatic management become more prominent gradually.

The use of mean rather than median survival explains the higher survival estimates used in our study (e.g., >12 years for Type 1 and >46 years for Type 2), compared to commonly reported median values [31, 32]. This approach better captures long-term survivors and provides a more comprehensive estimate of disease burden.

Our findings are consistent with previous studies showing a high economic and caregiver burden in SMA, particularly in more severe types [3-5]. A cross-sectional study conducted in KSA [13] showed that Type 1 SMA is associated with significantly higher hospitalization needs and that the disease imposes a considerable socioeconomic burden across all SMA types. Likewise, a cross-sectional study from Canada [4] highlighted the high caregiver burden, particularly in terms of psychological impact, which increases with disease severity.

This study provides country-specific estimates of the burden of SMA to support local decision-making. By quantifying the economic and humanistic impact within each national context, the study facilitates better interpretation by policymakers and healthcare stakeholders, thereby supporting informed reimbursement and resource allocation decisions.

Limitations

This study is subject to several limitations. First, as SMA is a rare disease, the small sample sizes in the available literature may affect the precision of estimated averages. Some model inputs were not available for the included countries; therefore, global estimates were used and adjusted where possible, including HUI3 values for disutility and time lost for patients and caregivers. To balance these limitations, we used local key parameters whenever available, including the following key parameters: prevalence, wages, labor force participation, employment rates, population life expectancy, and survival.

Second, there was no clear evidence on the number of caregivers per patient; however, indirect costs were based on total caregiving hours reported in the literature, making this parameter unnecessary for the analysis. Finally, although YLD estimates may be uncertain due to the use of HUI3 values instead of direct utilities, this was addressed through scenario analyses using a ±50% variance, providing a plausible range for DALYs.

Because comprehensive national data were not available for each country, the number of patients was estimated using epidemiological modelling. Some inputs were also based on international evidence or expert opinion. The results should therefore be viewed as informed estimates and interpreted in light of these uncertainties.

The strength of our study lies in its quantification of the disease burden for each specific country using local estimates, providing decision-makers with the country-specific evidence necessary to develop targeted, evidence-based health policies and resource allocations.

## Conclusions

SMA imposes a substantial impact on the QoL, survival, and financial stability of both patients and caregivers, driving a significant humanistic and economic burden. Effectively mitigating this burden through targeted interventions could improve patient survival, reduce the burden on healthcare budgets, and alleviate the overall societal impact of the disease.

## Figures and Tables

**Figure 2:**
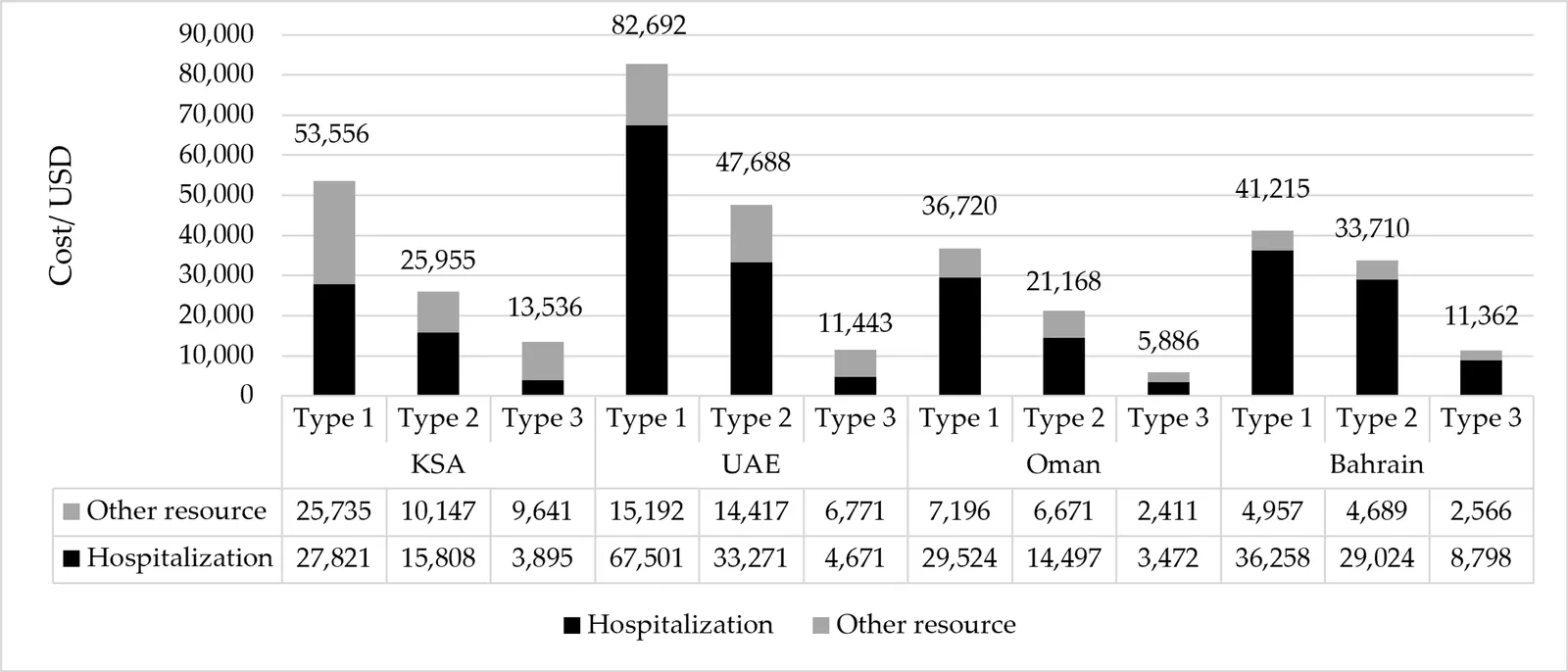
Direct annual costs per SMA patient SMA: Spinal Muscular Atrophy, KSA: Kingdom of Saudi Arabia, UAE: United Arab Emirates

## Appendices

Appendix A

**Table 6:** HUI3 values used as a proxy for utility of SMA subtypes. SMA: spinal muscular atrophy; HUI3: Health Utilities Index Mark 3.

SMA type	SMA Type 1 utility	SMA Type 2 utility	SMA Type 3 utility
Scenario 1 (50% higher utilities)	0.23	0.24	0.71
HUI3 reported values	0.15	0.16	0.47
Scenario 1 (50% lower utilities)	0.08	0.08	0.24

Appendix B

**Table 7:** Annual productivity lost by patients and their caregivers due to SMA in hours. SMA: spinal muscular atrophy.

	SMA Type 1 productivity lost annually (hours)	SMA Type 2 productivity lost annually (hours)	SMA Type 3 productivity lost annually (hours)
Patient	0	169	354
Caregiver	2,912	1,708	537

Appendix C

**Table 8:** Experts questionnaire: diagnostics.

Diagnostics	SMA Type I				SMA Type 2				SMA Type 3			
Percentages												
	Proportion	Frequency/ year	Cost/ visit	Total	Proportion	Frequency/ year	Cost/ visit	Total	Proportion	Frequency/ year	Cost/ visit	Total
EMG nerve conduction												
Genetic testing												

**Table 9:** Experts questionnaire: monitoring. CHOP INTEND: Children's Hospital of Philadelphia Infant Test of Neuromuscular Disorders; FEES: fibreoptic endoscopic evaluation of swallowing; DXA: dual-energy X-ray absorptiometry.

Monitoring	SMA Type I				SMA Type 2				SMA Type 3			
Proportion of patients monitored by SMA type: the percentage of patients being monitored at any point (what is the ratio of the patients you monitor?)												
	Proportion	Frequency/ year	Cost/ event	Total	Proportion	Frequency/ year	Cost/ event	Total	Proportion	Frequency/ year	Cost/ event	Total
Respiratory care: Monitoring												
Specialist visit (Pulmonologist)												
Full pulmonary function testing												
Respiratory Muscle Strength Study												
Monitoring gas exchange												
Respiratory Sleep Study (polysomnography)												
Oximetry or blood gas studies												
Hypoxic (Altitude) or Hyperoxic (Shunt) Assessment												
Cough effectiveness												
Lung volume studies												
Airflow studies												
Carbon monoxide transfer factor test												
Chest X-ray												
CT scan with contrast												
CT scan without contrast												
Echocardiogram												
Electrocardiogram												
Other 1												
Gastrointestinal care: Monitoring												
Evaluation of feeding and swallowing												
Gastroesophageal reflux assessment												
FEES												
Abdominal ultrasound												
Other 1												
Nutritional care: Monitoring												
Specialist visit (GIT)												
Calcium state test												
Vitamin D test												
Pre-albumin levels test												
Other 1												
Orthopedic care: Monitoring												
Specialist visit (orthopedist)												
Spinal X-ray												
MRI												
Bone density scan (DXA)												
X-ray hip												
Other 1												
Psychological: Monitoring												
Specialist visit (psychologist)												
Occupational therapy: Monitoring												
Occupational therapist visit												
Periodic musculoskeletal assessment (motor skills, CHOP INTEND)												
Musculoskeletal												
Visits												

**Table 10:** Experts questionnaire: management. SMA: spinal muscular atrophy; CPAP: continuous positive airway pressure; BiPAP: bilevel positive airway pressure.

Management	SMA Type I				SMA Type 2				SMA Type 3			
Proportion of patients treated by SMA type: the percentage of patients at treatment at any point (what is the ratio of the patients you treat?)												
	Proportion	Frequency/ year	Cost/ event	Total	Proportion	Frequency/ year	Cost/ event	Total	Proportion	Frequency/ year	Cost/ event	Total
Respiratory care (chronic/acute): Management												
Suction device												
Suction device (spare parts/ maintenance/ tubes)												
Cough assist device												
Cough assist device maintenance												
Antibiotics (courses)												
BiPAP												
CPAP												
Other noninvasive ventilation												
Tracheostomy												
Mechanical ventilation												
Vaccines												
Other 1												
Gastrointestinal care: Management												
Nasogastric tube placement												
Acid neutralizers												
Prokinetic agents												
Probiotics												
Other medications												
Laparoscopic anti-reflux procedure												
Gastrostomy												
Other procedures												
Nutritional care: Management												
Enteral/parenteral feeding												
Oral supplements												
Others												
Orthopedic care: Management												
Positioning and posture support												
Scoliosis surgery												
Others												
Others												
Musculoskeletal and Physiotherapy												
Physiotherapy												
AFO												
Spinal brace												
Seating devices												
Walking aids												
Wheelchair												
Other 1												

Appendix D
